# Patient-physician discrepancy in the perception of immune-mediated inflammatory diseases: rheumatoid arthritis, psoriatic arthritis and psoriasis. A qualitative systematic review of the literature

**DOI:** 10.1371/journal.pone.0234705

**Published:** 2020-06-17

**Authors:** José Antonio Sacristán, Tatiana Dilla, Silvia Díaz-Cerezo, Clara Gabás-Rivera, Susana Aceituno, Luis Lizán

**Affiliations:** 1 Medical department, Lilly Spain, Madrid, Spain; 2 Global Patient Outcomes and Real World Evidence, Lilly International, Madrid, Spain; 3 Outcomes’10, Castellón de la plana, Spain; 4 Department of Medicine, Jaume I University, Castellón de la plana, Spain; Universita Campus Bio-Medico di Roma, ITALY

## Abstract

**Introduction:**

Recommendations on chronic diseases management emphasise the need to consider patient perspectives and shared decision-making. Discrepancies between patients and physicians’ perspectives on treatment objectives, disease activity, preferences and treatment have been described for immune-mediate inflammatory diseases. These differences could result on patient dissatisfaction and negatively affect outcomes.

**Objective:**

To describe the degree of patient-physician discrepancy in three chronic immune-mediated inflammatory diseases (rheumatoid arthritis [RA], psoriatic arthritis [PsA] and psoriasis [Ps]), identifying the main areas of discrepancy and possible predictor factors.

**Methods:**

Qualitative systematic review of the available literature on patient and physician discrepancies in the management of RA, PsA and Ps. The search was performed in international (Medline/PubMed, Cochrane Library, ISI-WOK) and Spanish electronic databases (MEDES, IBECS), including papers published from April 1, 2008 to April 1, 2018, in English or Spanish, and conducted in European or North American populations. Study quality was assessed by the Oxford Centre for Evidence-Based Medicine criteria.

**Results:**

A total of 21 studies were included (13 RA; 3 PsA; 4 Ps; 1 RA, Ps, and Axial Spondyloarthritis). A significant and heterogeneous degree of discrepancy between patients and physicians was found, regarding disease activity, treatment, clinical expectations, remission concept, and patient-physician relationship. In RA and PsA, studies were mainly focused on the evaluation of disease activity, which is perceived as higher from the patient’s than the physician’s perspective, with the discrepancy determined by factors such as patient’s perception of pain and fatigue. In Ps, studies were focused on treatment satisfaction and patient-physician relationship, showing a lower degree of discrepancy in the satisfaction regarding these aspects.

**Conclusions:**

There is a significant degree of patient-physician discrepancy regarding the management of RA, PA, and Ps, what can have a major impact on shared decision-making. Future research may help to show whether interventions considering discrepancy improve shared decision-making.

## Introduction

Immune-mediated inflammatory diseases, including rheumatoid arthritis (RA), psoriatic arthritis (PsA) and psoriasis (Ps), are a group of chronic and highly disabling conditions that share common inflammatory pathways [1]. They affect millions of individuals worldwide, with a prevalence of 5%–7% in the Western society [1].

Recommendations for the treatment of chronic diseases emphasise the need to work in partnership with the patient [2]. For rheumatic diseases in particular, such as RA or PsA, the assessment of disease activity, as well as therapeutic decisions, rely heavily on patient-reported outcomes in combination with the physician’s perception, in contrast to other fields of medicine in which treatment decisions are based on measurable biomarkers [3]. Patient reported outcomes are reliable measures that allow the translation of qualitative clinical impressions into quantitative data [4,5]. They have shown to be as effective as physician-reported results or clinical variables in reflecting changes in disease activity over time [6]. In the case of dermatological diseases, such as Ps, several studies and clinical practice guidelines highlight the need to include both, the objective evaluation of severity and the subjective perception of the disease impact on the patient, for disease assessment [7], which is not routinely estimated [8].

In clinical practice, patient and physician perspective regarding disease state and treatment expectations may differ. Discrepancies in the assessed health status may result in patient dissatisfaction and could negatively affect patient care, treatment compliance and disease outcomes, with the consequent cost to society [2,9].

The literature about the differences between patients and physicians’ perspectives regarding the management of their disease is heterogeneous, and the potential predictors of the discrepancy are not clear [2]. The objective of this systematic review is to describe the degree of discrepancy between patients and physicians in the management of three immune-mediated inflammatory diseases (RA, PsA and Ps), identifying the main areas of discrepancy and the possible predictor factors.

## Methods

A qualitative systematic review of the literature on the existing differences between patients and physicians’ perspective in the management of RA, PsA and Ps was conducted according to PRISMA recommendations [10].

To identify relevant articles, targeted literature searches in international (Medline/PubMed, Cochrane Library, ISI Web of knowledge [ISI WOK]) and Spanish electronic databases (Medicina en Español [MEDES], Índice Bibliográfico Español en Ciencias de la Salud [IBECS]) were conducted. The search strategy was focused on the conditions of interest, the physician and patient figures, and terms related to discrepancy between them (S1 Table). Reference lists of the selected articles were hand-searched to identify additional potentially relevant publications.

The search was limited to studies published in English or Spanish, from April 1, 2008 to April 1, 2018. Studies that focused on the discrepancy between patients and physicians in the management of RA, PsA and Ps, conducted in Europe or North America (or international publications comprising European or North American populations) were included. We excluded congress abstracts, study protocols, letters to the editor and those publications reviewed in the systematic review included in our review.

After removal of duplicates, the records were screened by two independent researchers, in two levels. The first level included title or abstract screening, and the second level included full text screening. Discrepancies were reviewed by a third researcher and resolved by consensus. The included studies were graded on quality of evidence according to the Oxford Centre for Evidence-Based Medicine (OCEBM) criteria [11], where level 1 is the maximum level of evidence (e.g. systematic reviews) and level 5 is the minimum level of evidence (e.g. expert opinion). Risk of bias was measured though a Newcastle-Ottawa scale adapted for cross-sectional studies [12,13], where the maximum score is 10, and the minimum is 0, considering a low risk of bias 7 points or more, and high risk of bias 6 points or less. Risk of bias for systematic reviews was measured through ROBIS tool, which classifies the studies in high, low or unclear risk of bias [14].

General data were extracted from each publication, including country, design, disease studied, participants, and the level of evidence. The variables of discrepancy were identified in each publication (discrepancy definition, measurement tools, and results) and grouped according to the disease. Predictor factors of discrepancy (measurement tools and results) were summarized for each publication.

## Results

**Fig 1:**
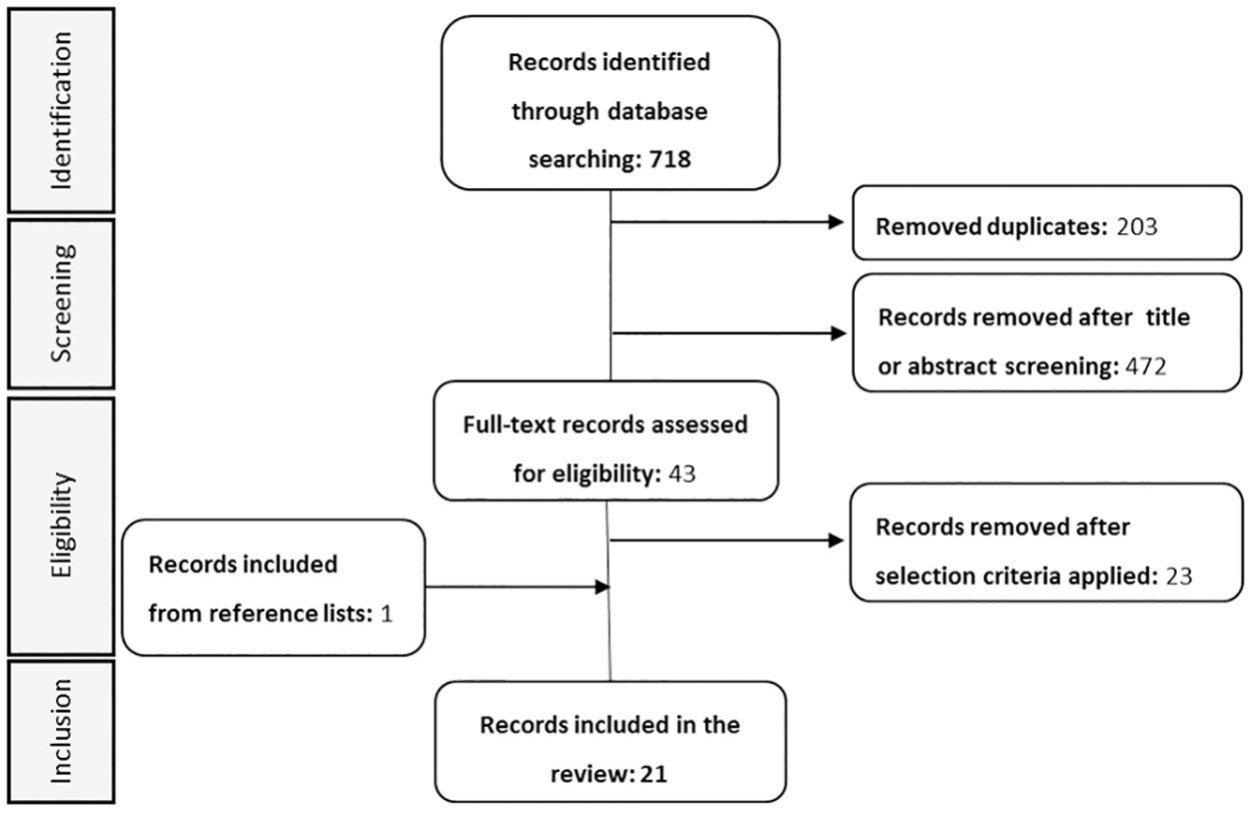
Study selection flowchart according to PRISMA.

A total of 718 titles were identified. Of them, 20 publications were relevant to the objective of the study and, therefore, were selected. An additional record from references’ review was identified, hence 21 articles were finally included in the review (Fig 1). The general characteristics of the 21 studies reviewed are summarized in Table 1. Main results (discrepancy and predictor factors) for each study are shown in Table 2, and described further (discrepancy definition, measurement tools, detailed results) in S2 Table. Excluded publications based on the selection criteria are shown in S3 Table.

**Table 1:** Characteristics of the 21 studies included in the systematic review.

Author, year	Country	Study design	Discrepancy area	Study participants	Level of evidence
**RHEUMATOID ARTHRITIS**					
**Acebes et al.** [15], **2017**	Spain	Cross-sectional	Remission concept	5 RA patients	5
				18 rheumatologists (6 involved in basic research, 6 with high specialisation in imaging techniques and 6 clinical rheumatologists)	
**Challa et al.** [16], **2017**	USA	Cross-sectional	Disease activity assessment	350 patients with RA or rheumatoid polyarthritis	2c
				Healthcare professionals (physician, fellow, nurse practitioner, physician assistant) *	
**De Mits et al.** [17], **2016**	Belgium	Cross-sectional	Treatment	550 RA patients	2c
				67 rheumatologists	
**Desthieux et al.** [2], **2016**	France	Meta-analysis of international studies	Disease activity assessment	12 studies including 11.879 RA patients Physicians*	2a
**Janta et al.** [18], **2013**	Spain	Prospective and cross-sectional	Disease activity assessment	69 RA patients in clinical remission according to rheumatologist	2c
				1 rheumatologist	
**Karpouzas et al.** [19], **2017**	USA	Prospective (2-year follow up)	Disease activity assessment	536 patients with stable RA Rheumatologists*	2c
**Kvrgic et al.** [20], **2017**	USA	Cross-sectional	Disease activity assessment	20 RA patients with patient-physician discrepancy	5
**Markenson et al.** [21], **2013**	USA	Retrospective	Treatment	4.359 RA patients Rheumatologists*	2c
**Smolen et al.** [22], **2016**	International: Europe, Asia, Australia, Latin America	Retrospective (36-weeks follow-up)	Disease activity assessment	763 RA patients Physicians*	2c
**Walter et al.** [23], **2017**	Netherlands	Cross-sectional	Disease activity assessment	29 RA patients with patient-physician discrepancy	5
**Ward et al.** [24], **2017**	USA	Prospective (4-month follow-up)	Disease activity assessment	206 patients with active RA	2c
				4 rheumatologists	
**Wen et al.** [25], **2012**	International: USA, China, Japan	Cross-sectional	Clinical visit expectations	270 RA patients	4
				111 physicians	
**Wolfe et al.** [26], **2009**	USA	Cross-sectional	Disease activity assessment	800 RA patients	4
				Rheumatologists*	
**PSORIASIS**					
**Daudén et al.** [27], **2011**	Spain	Cross-sectional	Treatment Patient-Physician Relationship	771 Ps patients	2c
				151 dermatologists	
**Gonzalez et al.** [28], **2016**	United Kingdom	Cross-sectional	Treatment	174 patients	2c
				100 dermatologists	
**Korman et al.** [29], **2016**	USA	Retrospective	Treatment	627 paired dermatologists and Ps patient records	2c
**Uhlenhake et al.** [30], **2010.**	USA	Cross-sectional	Patient-Physician Relationship	25 Ps patients	5
				29 dermatologists	
**PSORIATIC ARTHRITIS**					
**Desthieux et al.** [31], **2017**	Europe (13 counties involved)	Cross-sectional	Disease activity assessment	460 PsA patients	2c
				Physicians*	
**Eder et al.** [3], **2015**	Canada	Cross-sectional	Disease activity assessment	565 PsA patients	2c
				Rheumatologists*	
**Furst et al.** [32], **2017**	USA	Retrospective	Disease activity assessment	305 paired rheumatologists and PsA patient records	2c
**RHEUMATOID ARTHRITIS, PSORIATIC ARTHRITIS AND AXIAL SPONDYLOARTHRITIS**					
**Lindström Egholm et al.** [33], **2015**	Denmark	Retrospective	Disease activity assessment	10.282 patients (8.300 RA patients, 1.458 PsA patients and 524 axSpA patients)	2c
				90 physicians (50% were specialists)	

* Number of physicians not specified; ^ Quality of evidence graded according to the Oxford Centre for Evidence-Based Medicine criteria

**Table 2:** Discrepancy and predictor factors in rheumatoid arthritis, psoriatic arthritis and psoriasis.

Author, year	Results
**RHEUMATHOID ARTHRITIS. Discrepancy area: DISEASE ACTIVITY ASSESSMENT**	
**Karpouzas et al.** [19], **2017**	Discrepancy: **43%** (PD: 31.3%; ND: 11.7%).
	Predictor factors. PD: higher fatigue, pain, HAQ-DI, lower TJC and SJC, and worse GH (p<0.02); ND: lower pain, higher TJC and SJC and PHQ-9 (p<0.01).
**Challa et al.** [16], **2017**	Discrepancy: **32.5%** (PD: 29.5%; ND: 2.1%).
	Predictor factors (OR, 95% CI): Diagnosis of fibromyalgia 3.06 (1.87–8.00); depression 1.79 (1.02–3.15); lack of articular erosions 0.56 (0.32–0.97).
**Desthieux et al.** [2], **2016**	Discrepancy: **43%** (95% CI: 36%-51%; range: 25%-76%); (PD: 34%; ND: 9%).
	Predictor factors: pain; TJC/SJC; higher levels of depressive symptoms; health literacy.
**Ward et al.** [24], **2017**	Discrepancy: lower with rating scale vs. PhGA (p<0.0001) (**PtGA—PhGA: 8.5** ± 22.4; **Rating scale—PhGA: 2.3** ± 24.0)^
**Smolen et al.** [22], **2016**	General patients:
	Discrepancy. **Baseline: PD 25.5%**, ND: 6.3%; **week 36** of etanercept + methotrexate treatment: **PD 24.8%**, ND: 2.4%.
	Predictor factors:
	Baseline factors correlated with 36-week discrepancy (r<0.25, p<0.05). Directly correlated: BPI, duration of morning stiffness and GH; Inversely correlated: fatigue, and SJC.
	Factors measured in week 36. Moderate correlation: BPI, GH (r = 0.48 y r = 0.58, respectively, p<0.0001). Weak correlation (r<0.25, p<0.0001): directly correlated: DAS28, duration of morning stiffness, HAQ-DI, CDAI y SDAI; inversely correlated: fatigue.
	Factors measured at baseline predicting the discrepancy at week 36 (OR, 95% CI): BPI 1.22 (1.11–1.35), CRP 0.98 (0.97–1.00) and GH 1.02 (1.00–1.03).
	Discrepancy in remission patients. Remission according to clinical and Boolean criteria (PD: 0%; DN: 2.0%); according to clinical but not Boolean criteria (PD: 49.2%; DN: 1.1%); according to CDAI (PD: 7.8%; DN: 1.0%).
**Wolfe et al.** [26], **2009**	Patients discordant with their physicians: **21.4%**; patients concordant with their physicians: 78.6% (K: 0.54, 95% CI: 0.45–0.58)
**Janta et al.** [18], **2013**	Discrepancy regarding the percentage of **patients in remission**. According to **DAS28: 26.1% (patients) vs. 52.2% (physicians)** (p<0.0005); according **SDAI: 14.5% (patients) vs. 11.6% (physicians)** (p = 0.172)
**Kvrgic et al.** [20], **2017**	Predictive factors of discrepancy from patients’ perspective: Being misunderstood by others; Limitations of physician assessments; Discrepancy with physicians’ findings; Inadequate active listening by doctors; Unmet psychosocial needs; Lack of patient empowerment during clinical visits.
**Walter et al.** [23], **2017**	Predictive factors of discrepancy (PD) in disease activity from patients’ perspective: 1) perceived stress, 2) balancing activities and rest, 3) medication intake, 4) social stress, 5) relationship with professionals, 6) comorbidity, and 7) physical fitness.
**RHEUMATHOID ARTHRITIS. Discrepancy area: TREATMENT**	
**De Mits et al.** [17], **2016**	Satisfaction with **symptom control: 44% of satisfied patients vs. 35% of satisfied physicians** [OR = 3.9 (2.6 ± 5.8); p < 0.001].
	Satisfaction with **route of administration. IV route: 52.4% of satisfied patients vs. 29.9% of satisfied physicians** (p<0.001); **SC route: 56.2% of satisfied patients vs. 45.5% of satisfied physicians** (p<0.001).
**Markenson et al.** [21], **2013**	**Baseline**: similar **PtGA and PhGA** scores (**5.90 vs. 5.85**); **5 years** follow-up: PtGA higher than PhGA (between **4.05–4.46 for PtGA vs. 2.74–3.76 for PhGA**)^.
**RHEUMATHOID ARTHRITIS. Discrepancy area: CLINICAL VISIT EXPECTATIONS**	
**Wen et al.** [25], **2012**	Patient’ main expectations: pain control (63.7%); improvement of function (49.3%); discussion about the effect of medication (38.1%). Physician’ main expectations: pain control (59.5%), inquiry about drug side-effects (47.8%); objective assessment of disease activity (41.4%).
**RHEUMATHOID ARTHRITIS. Discrepancy area: REMISSION CONCEPT**	
**Acebes et al.** [15], **2017**	Rheumatologists: highlighted quantifiable objective parameters.
	Patients: preferred subjective measures of remission (need of finding a new definition of remission, new assessment tools that consider their feelings and all the symptoms they suffer).
**PSORIATIC ARTHRITIS. Discrepancy area: DISEASE ACTIVITY**	
**Eder et al.** [3], **2015**	Discrepancy. **Joint activity: 32.8%** (PD: 31.2%, ND: 1.6%); **Skin activity: 22.2%** (PD: 15.4%, ND: 6.8%).
	Predictor factors. Joint activity: fatigue (21.3%), TJC (16.3%), pain (9.2%), and SJC (1.5%); Skin activity: pain (17.3%), DLQI (14%) and PASI (11.8%).
**Desthieux et al.** [31], **2017**	Discrepancy: **29.1%** (PD: 25.0%, ND: 4.1%); Discordant patients in remission: 30.8%; discordant patients with high disease activity: 26.1%
	Predictor factors (higher discrepancy): higher fatigue, lower self-perceived coping and impaired social participation.
**Furst et al.** [32], **2017**	Discrepancy: **23.6%** (satisfied patient- dissatisfied physician: 17.0%; dissatisfied patient—satisfied physician: 6.6%)
	Predictor factors: SJC (p = 0.020), HAQ-DI (p = 0.025)
**PSORIASIS. Discrepancy area: TREATMENT**	
**Korman et al.** [29], **2016**	Discrepancy: **18,4%** (in 70.4% of cases, patient was satisfied, and physician dissatisfied).
**Daudén et al.** [27], **2011**	**No significant discrepancies** on treatment satisfaction and treatment compliance between physicians and patients were observed (p>0,05).
**Gonzalez et al.** [28], **2016**	Discrepancies: 1) Improvements in plaques on limbs were more important than plaques on the torso for physicians, but not for patients; 2) Patients perceived a significant benefit in reducing mild plaque area from 10% to 0%, but not physicians; 3) Patients perceived the impact of an area of 10% very severe plaques to be much more important than dermatologists; 4) Dermatologists valued improvements in very severe plaques for areas greater than 10%, but patients were insensitive to changes in the affected area beyond 10%; 5) Dermatologists were more sensitive to 10% lymphoma risk in the next 10 years than patients.
	Maximum Acceptable Risk: higher in patients than in physicians.
**PSORIASIS. Discrepancy area: PATIENT-PHYSICIAN RELATIONSHIP**	
**Daudén et al.** [27], **2011**	**No significant differences** were observed: 1) Almost all patients and physicians considered their relationship was good or very good (96.4% vs. 96%, respectively); 2) Patients had a good opinion about the physician (98% vs 95.2%); 3) Patients were satisfied with the treatment received (92% vs 94.7%); 4) Patients were satisfied with the time spent by the specialist (97.1% vs 92.2%).
**Uhlenhake et al.** [30], **2010**	Patients required more information about Ps, fast-acting treatments, clear expectations, and recognition of the emotional burden.
	Physicians considered that patients do not internalize information adequately and need more information about treatments.
**RHEUMATOID ARTHRITIS, PSORIATIC ARTHRITIS AND AXIAL SPONDYLOARTHRITIS. Discrepancy area: DISEASE ACTIVITY ASSESSMENT**	
**Lindström Egholm et al.** [33], **2015**	Discrepancy. **RA: 49%** (PD: 47.1%, ND: 1.9%); **PsA: 56.5%** (PD: 56.2%, ND: 0.3%); axSpA: 48.3% (PD: 46.9%, ND: 1.4%)
	Predictor factors (higher discrepancy). RA (higher discrepancy): patient female sex, older age, lower SJC and higher TJC, higher CRP, treatment with biologics; PsA: lower SJC and higher TJC; AxSpA: patient female sex, treatment with biologics

PD: positive discrepancy; ND: negative discrepancy; PtGA: patient global assessment; PhGA: physician global assessment; DAS: Disease Activity Score; HAQ-DI: Health assessment questionnaire disability index; TJC: tender joint count; SJC: swollen joint count; GH: general health; CDAI: Clinical Disease Activity Index; SDAI: Simple Disease Activity Index; BPI: Brief Pain Inventory; CRP: C-Reactive Protein; CI: confidence interval; IV: intravenous; SC: subcutaneous; DLQI: Dermatology Life Quality Index; PASI: Psoriasis Area Severity Index; RA: Rheumatoid Arthritis; PsA: Psoriatic Arthritis; axSpA: Axial Spondyloarthritis; OR: odds ratio. ^Higher PtGA and PhGA denote worse assessments.

### Characteristics of the selected studies

#### Disease

Thirteen (62%) articles evaluated the differences between patients and physicians’ perspectives on RA, 3 (14%) on PsA, and 4 (19%) on Ps. One article (5%) included patients with different diseases (RA, PsA, and Axial Spondyloarthritis [axSpA]).

#### Country

Forty-three percent of the studies (n = 9) included were conducted in the USA, 38% (n = 8) in Europe (Belgium, Netherlands, Denmark, Spain, UK and European Union), 5% (n = 1) in Canada, and 14% (n = 3) were conducted internationally.

#### Design

Ninety-five percent of the studies (n = 20) were observational. Of these, 24% (n = 5) followed a retrospective, 14% (n = 3) a prospective, and 57% (n = 12) a cross-sectional design. The remaining publication (5%) was a systematic review with meta-analysis.

#### Level of evidence and risk of bias

Using the Oxford Centre for Evidence-Based Medicine criteria, the quality of evidence was graded. Sixty-seven percent of the studies were graded 2c (n = 14), 5% (n = 1) of studies had level 2a, 10% (n = 2) level 4, and the remaining 19% (n = 4) had level 5. Therefore, the included studies would have a B-D recommendation grade.

Risk of bias measured through Newcastle-Ottawa scale showed that 62% (n = 13) of the studies had low risk of bias (7 points or more), and 33% (n = 7) had a high risk of bias (6 points or less), with four of those studies being qualitative. The remaining 5% (n = 1) was measured with ROBIS scale for systematic reviews and showed a low risk of bias (S4 Table and S1 Fig).

#### Discrepancy area

Sixty-two percent of the studies (n = 13) focused on disease activity, 24% (n = 5) on treatment, 10% (n = 2) on patient-physician relationship (one of the articles evaluated discrepancies in both treatment and patient-physician relationship), 5% (n = 1) focused on the clinical visit expectations, and 5% (n = 1) on the concept of remission.

### 1. Rheumatoid arthritis

#### Disease activity assessment

Sixty-nine percent (n = 9) of the RA studies included evaluated the discrepancies in disease activity assessment [2,16,18–20,22–24,26].

Five of them included RA general patients and used the Patient and Physician Global Assessments (PtGA and PhGA, respectively) questionnaires [2,16,19,22,24]. The degree of discrepancy found in these studies ranged between 25% [2] and 76,0% [2]. The majority of patients reported higher values than physicians in the overall assessment, indicating a greater perception of disease activity from the patient’s perspective (defined as positive discrepancy, PD). On the other hand, to a lesser extent, cases of negative discrepancy (ND) were described, where the physician’s assessment indicated higher disease activity than the patient’s self-assessment. Four studies evaluated the predictor factors of patient-physician discrepancy [2,16,19,22], finding that the discrepancy observed was mainly influenced by the Tender Joint Count (TJC) and Swollen Joint Count (SJC), pain, fatigue, general health (GH) and the onset of depressive symptoms. Some studies described a variation of the discrepancy, either according to the patients’ disease activity or over time. Two studies showed poorer agreement in patients with higher RA activity, compared to those in remission or with low RA activity [16,24]. Another one reported a decrease in discrepancy over time (from 31.8% at baseline to 27.2% at 36 weeks of treatment) and found an association between higher discrepancy and worse clinical outcomes (TJC, SJC, pain, Clinical Disease Activity Index [CDAI], Simple Disease Activity Index [SDAI], GH) [22] (Table 2). Additionally, one study examined the impact of PD and its persistence on health-related quality of life (HRQoL) and work productivity on final visit, finding that higher patient ratings (PD) at any time in the study (baseline visit, 1 year, 2 years) were associated with worse HRQoL, work productivity and activity impairment on final visit, compared with patients who did not present PD [19].

Three out of the nine studies evaluated patient-physician discrepancy in disease activity assessment in RA patients in remission [18,22,26]. Although the procedures used and the definition of remission varied among the studies, results showed that, in general, patients and physicians had different perceptions regarding disease activity. Thus, the number of patients in remission was lower from the patient perspective than from the physician perspective (Table 2).

Two out of the nine studies explored the factors that patients considered relevant for the PD in disease activity [20,23]. Through focus groups interviews, seven themes came out: perceived stress, balancing activities and rest, medication intake, social stress, relationship with professionals, comorbidity, and physical fitness [23]. From patient interviews, six major themes emerged: being misunderstood by others, limitations of provider assessments, discrepancy with provider findings, inadequate active listening on the part of health care providers, unmet psychosocial needs, and lack of patient empowerment [20] (Table 2).

#### Treatment

Fifteen percent (n = 2) of the RA studies evaluated the discrepancies regarding treatment.

One of them assessed patient and physician satisfaction with biological medications in relation to the control of disease symptoms and the route of administration [17]. The results showed a higher patient satisfaction with both symptom control and route of administration, compared to physicians (p<0.001), regardless of the route of administration. The physician’s perception of patient’s satisfaction with disease control was markedly lower for intravenous treated patients as opposed to subcutaneous treated patients (p< 0.001). The second study evaluated the discrepancy in the perceived therapeutic effectiveness of Disease Modifying Antirheumatic Drugs (DMARD). PtGA and PhGA were similar at baseline, but in the follow-up assessments, a worse evaluation for DMARD effectiveness was found from the patients’ perspective, compared to the physicians [21].

#### Clinical visit expectations

Eight percent (n = 1) of the RA studies focused on patient-physician discrepancy over the expectations during the clinical visit [25]. The study compared the expectations of patients and physicians about what was most important to achieve during a rheumatology clinic visit. Both agreed on their main expectation: pain control. Expectations from the patients’ perspective were pain control (63.7%), improvement of function (49.3%) and discussion of effect of medication (38.1%). From physicians’ perspective, the main expectations were pain control (59.5%), inquiry about drug side-effects (47.8%) and objective assessment of disease activity (41.4%). The main difference between patients and physicians was in the importance of the objective assessment of disease activity, which was prioritized by physicians, but not by patients.

#### Remission concept

Eight percent (n = 1) of the RA studies focused on patient-physician discrepancy over the concept of remission itself [15]. The results showed a discrepancy between patients and physicians, as well as among physicians themselves. Rheumatologists highlighted quantifiable objective parameters, while patients preferred subjective measures of remission, pointing out the need of finding a new definition of remission and new assessment tools that consider what they feel and the wide range of symptoms they suffer. Nonetheless, many rheumatologists agreed with patients that a new definition of remission should consider two concepts: psychosocial variables and the context. On the other hand, physicians disagreed among themselves on the value given to the different parameters to diagnose remission. While some of them were in favour of seeking an objective definition of remission with the assistance of some type of instrument, others safeguarded a subjective point of view in which each patient has his own point of disease remission.

### 2. Psoriatic arthritis

Three of the articles included in the review evaluated the differences between patients and physicians’ perspectives on PsA regarding disease activity [3,31,32]. Two of them focused on disease activity in general [3,31], while the third one evaluated the discrepancy over the satisfaction with the control of disease activity [32]. The three studies evaluated additionally the predictor factors or factors affecting the discrepancy.

Results revealed a PD (patients indicated more severe disease) between patients and physicians. This discrepancy was greater for the assessment of joint activity (31.2%) than for skin activity (15.4%). To a lesser extent, a ND (patients indicated more less severe disease) was observed: 1.6% in the assessment of the joints and 6.8% for the skin [3]. Discrepancy was greater in patients in remission (30.8%), compared to patients with high disease activity (26.1%) [31]. In general, PsA patients were satisfied with the control of their disease activity. Patient-physician discrepancy regarding this satisfaction was 23.6%, mainly explained by the dissatisfaction of physicians with the control of their patients’ disease [32]. Misaligned patients reported greater work impairment, assessed with Work Productivity Activity Impairment index (work impairment, mean 38.7 vs. 21.4, P = 0.0004; presentism, mean 36.2 [25.3] vs. 16.5 [21.2], P < 0.0001; and daily activities, mean, 38.7 vs. 22.3, P < 0.0001), and higher disease burden (mean Health Assessment Questionnaire-Disability Index [HAQ-DI] 0.56 vs. 0.37, P = 0.0001), compared to those patients aligned with their physicians [32].

Fatigue and pain were the main predictor factors of the discrepancy between patients and physicians [3,31]. A higher SJC and HAQ-DI score predicted greater patient-physician discrepancy in relation to satisfaction with disease control [32].

### 3. Psoriasis

Four of the articles selected in the review evaluated the differences between patients and physicians perspectives on Ps [27–30].

#### Treatment

Seventy-five percent (n = 3) of Ps studies evaluated the discrepancies regarding treatment [27–29]. One of them focused exclusively on treatment satisfaction [29], another on treatment satisfaction and compliance [27], and the third evaluated patient-physician preferences for the outcomes of Ps treatments [28].

Patient-physician discrepancy regarding satisfaction with the treatment for Ps was 18.4%, where in 70.4% of the cases the physicians were dissatisfied, and patients were satisfied. Misalignment was associated with increased disease and symptom severity, poorer HRQoL, and reduced work productivity. Additionally, patients in the misaligned group were, on average, more recently diagnosed than those in the aligned group [21].

Discrepancy regarding compliance with treatment was not present. The perception of patient HRQoL affectation was very similar between patients and physicians, with no discrepancy between them. According to dermatologists, 56.4% of patients had a high or moderate physical impairment and 63.8% had a high or moderate emotional impairment; while these assessments made by the patients amounted to 56.5% and 60.5%, respectively [27].

In reference to treatment preferences, patients and physicians differed in which symptoms they preferred to improve and in the importance of the risk of adverse events. Dermatologists perceived improvements in plaques on limbs were more important than plaques on the torso, while there were no differences in patients. However, patients perceived a significant benefit in reducing mild plaque area from 10% to 0%, while dermatologists didn’t. Similarly, an area of 10% of very severe plaques had a greater impact for patients than for dermatologists. Dermatologists valued improvements in very severe plaques for areas greater than 10%, while patients were insensitive to changes in the affected area beyond 10%, but were more sensitive to a 10% lymphoma risk in the next 10 years than patients. Additionally, compared to physicians, patients were generally more willing to assume a risk of adverse events in exchange for clinical benefits, although this difference was only significant for clearance of very severe plaques [28].

#### Patient-physician relationship

Fifty percent (n = 2) of Ps studies assessed the discrepancies in patient-physician relationship [27,30], being one of them focused on communication issues [30]. An agreement between patients and physicians was found regarding their perception of the relationship with the doctor, opinion about the doctor, satisfaction with the treatment received by the doctor, and satisfaction with the time the doctor dedicates to the patient [27]. However, a disagreement was found in patient-physician communication regarding compliance issues, treatment plan preferences and goals, education, and emotional burden [30].

### 4. Rheumatoid arthritis, psoriatic arthritis and axial spondyloarthritis

One of the studies included in the review evaluated the frequency of patient-physician discrepancy over disease activity assessment in patients with RA, PsA or axSpA, and investigated whether a greater discrepancy in female patients (compared to male patients) was associated with physicians’ gender [33].

Results showed a patient-physician discrepancy in approximately 50% of cases. Male patients had lower odds of discordance compared with female patients across all 3 diagnoses, although not statistically significantly in PsA. Lower SJC and higher TJC increased the odds of discrepancy in RA and PsA. Older patients with RA had slightly higher odds of discordance. Patients who were not treated with biologicals tended to have lower odds of discordance.

## Discussion

Compared to other diseases, such as diabetes or hypertension, where an objective and numerical measurement to assess disease severity and treatment response is available (hemoglobin A1c or blood pressure), the lack of a single gold standard measurement to assess disease activity in immune-mediated inflammatory diseases difficult its management, pointing out the need to consider both objective and subjective assessments [34]. Moreover, taking into account patients perspective and involving them in disease activity assessment may enhance self-management behaviour and ultimately improve health outcomes [35].

The studies reviewed show that patients and physicians focus on different aspects of the disease, resulting in different perceptions of disease severity, diverse clinical expectations or different impressions of treatment response. In immune-mediated inflammatory diseases, these discrepancies are associated with worse clinical outcomes, activity impairment, reduction in work productivity and poorer HRQoL [19,21,22,32]. Previous studies have reported that discordance is also associated with a lower likelihood of remission in patients with RA and PsA [36]; greater joint destruction and functional impairment in RA [37], and higher Disease Activity Score (DAS28) and C-Reactive Protein (CRP) after the 24 weeks of disease-modifying therapy in early RA [38]. This association between discrepancy and worse clinical outcomes has also been described in other diseases such as asthma [39].

This systematic review denotes that the literature addressing patient-physician discrepancy in RA, PsA and Ps is very heterogeneous and highlights the lack of a standardized criterion to define discrepancy, with patients and physicians’ perspectives appraised using diverse tools, and discrepancy established according to different cut-off values. This circumstance results in different degrees of discrepancy according to the criteria considered and makes it difficult to compare results between studies. In this regard, an inverse correlation between the frequency of discrepancy and the cut-off point used has been previously reported in the literature, being the discrepancy higher when the cut-off point is lower [2].

Differences among immune-mediated inflammatory diseases have been detected. Thus, the studies conducted in RA and PsA were mainly focused on the evaluation of disease activity, while studies performed in Ps were focused on treatment satisfaction and preferences, and on patient-physician relationship.

In RA, PtGA-PhGA discrepancy about disease activity assessment varied among studies, ranging from 25% to 76%. In general, it was lower in patients in remission, and greater in patients with moderate or high disease activity. Patient and physician disagreement has been previously reported [34,40–42] and, in many cases, may adversely affect therapeutic decisions [43] and the assessment of treatment response [41]. The discrepancy could be explained in part, since patients could rely more heavily on the subjective perception of pain and discomfort, and therefore discrepancy will not only reflect the disease status but also psychological distress and comorbidities [21,44]. Additionally, another study in RA concluded that physicians underestimated disease severity and treatment related adverse events and their impact on patient perceived well-being [45]. Physicians are generally more prone to use objective measures to determine treatment response and may not pay sufficient consideration to patient-reported variables [21]. Accordingly, expectations during the clinical visit also differed on the objective assessment of disease activity in RA, which was prioritized by physicians, but not by patients, although both, patients and physicians, shared the same objective: pain control [25]. Previous studies have reported that RA patients have higher expectations from their treatment than physicians, specifically in terms of pain control [46]. Finally, in relation to satisfaction with disease treatment, RA patients receiving biologics generally revealed better satisfaction about the control of the symptoms, regardless of the route of administration, while physicians consistently considered IV biological therapy to be less satisfactory. Even though the factors associated with the discrepancy in RA have not been well established [47], our results show that major factors affecting PtGA-PhGA discrepancy in disease activity assessment are TJC, SJC, fatigue and pain. The awareness of which factors contribute to physicians’ and patients’ perceptions may help to define an improved standard measurement to better assess disease activity and treatment response, and to establish an enhanced patient-physician dialogue [21,47].

In line with previous studies conducted in PsA [21], a greater PD for joint activity (31.2%) compared to skin activity (15.4%) was observed. This discrepancy was predicted mainly by fatigue and pain. At present, there is an increasing trend to rely on patient self-reported questionnaires of disease activity and treatment response for monitoring patient status and adjusting treatment if necessary [35]. Therefore, according to our results, as patient self-report does not agree with physician observation, to rely solely on patient self-reported joint counts may not be appropriate [48], or vice-versa, suggesting the need to use both objective and subjective measurements. Our results indicate that, contrary to RA, discrepancy was greater in patients in remission, compared with high disease activity patients.

In Ps, low discrepancies were detected related to treatment satisfaction in Ps or patient physician relationship. Patient physician relationship is key to achieve a high-quality health care, as it has been described that many doctors tend to overestimate their ability in communication and that much patient dissatisfaction and complaints are due to breakdown in the doctor-patient relationship [49]. Nonetheless, some patient-physician differences were found regarding treatment preferences, objectives, compliance or emotional burden. Accordingly, despite previous communications reporting different perceptions between psoriasis patients and their physicians with respect to disease severity, symptoms, disease control [50] or treatment goals [51], the results of this review suggest improvement in the dialogue between patient and physician.

This qualitative systematic review has several limitations. The first is related to the search strategy, as it does not include all possible databases (including Embase) and the grey literature, which might imply a incompleteness of the results. Secondly, the search was limited to studies published from 2008 onwards, as it is in the last decade, with the introduction of biological drugs, where the management and treatment of immune-mediated inflammatory diseases has experienced a major change. Similarly, it was limited to studies conducted in Europe and North America and published in English and Spanish. Thirdly, the heterogeneity of the articles included, in terms of diseases, population and methodology could represent a limitation. As a whole, there was a lack of uniformity regarding the measurement tools and thresholds established to formally assess the patient-physician discrepancy. The quality of the studies included is moderate, what is anyway inherent to the topic of the literature search. In this regard, a review considering different selection criteria could generate different conclusions. Nonetheless, the results of this systematic review will allow a better understanding of the areas and the degree of discrepancy as well as to the determinants that contribute to the discrepancy between patients and physicians. It is anticipated that a better understanding of these factors can lead to the development of better strategies for the improvement of immune-mediated inflammatory diseases management.

In conclusion, this systematic review reveals a significant patient-physician discrepancy in RA and PsA, being lower for Ps. Based on these results, the physician assessment should be complemented with a self-report from patient’s perspective, which may facilitate patients involvement in the management of their disease. This collaborative approach between patient, physician, and other health professionals can contribute to patient trust in the physician, allowing patients to express their concerns and thoughts, prioritize their problems, and discuss with the physician their expectations and goals, what could improve patient outcomes and increase adherence to treatment. Future research may help to show whether identifying the discrepancy between patients and physicians, and a better knowledge of the factors that influence it, may contribute to improve patient care and shared decision-making.

## Supporting information

S1 Fig Assessment of Desthieux et al., 2016 study using ROBIS scale for systematic reviews. (DOCX)

S1 Table Terms and search strategy in international and Spanish databases. (DOCX)

S2 Table Discrepancy and predictor factors in immune-mediated inflammatory diseases (RA, PsA and Ps). (DOCX)

S3 Table Articles excluded. (DOCX)

S4 Table Risk of bias of the selected studies using the Newcastle-Ottawa scale, adapted for cross-functional studies. (DOCX)
