# Supplementary figures and images for: Patient-physician discrepancy in the perception of immune-mediated inflammatory diseases: rheumatoid arthritis, psoriatic arthritis and psoriasis. A qualitative systematic review of the literature
Source: PLoS One. 2020 Jun 17;15(6):e0234705. doi: 10.1371/journal.pone.0234705 (PMC7299355; doi:10.1371/journal.pone.0234705)

Supplementary Figure S1


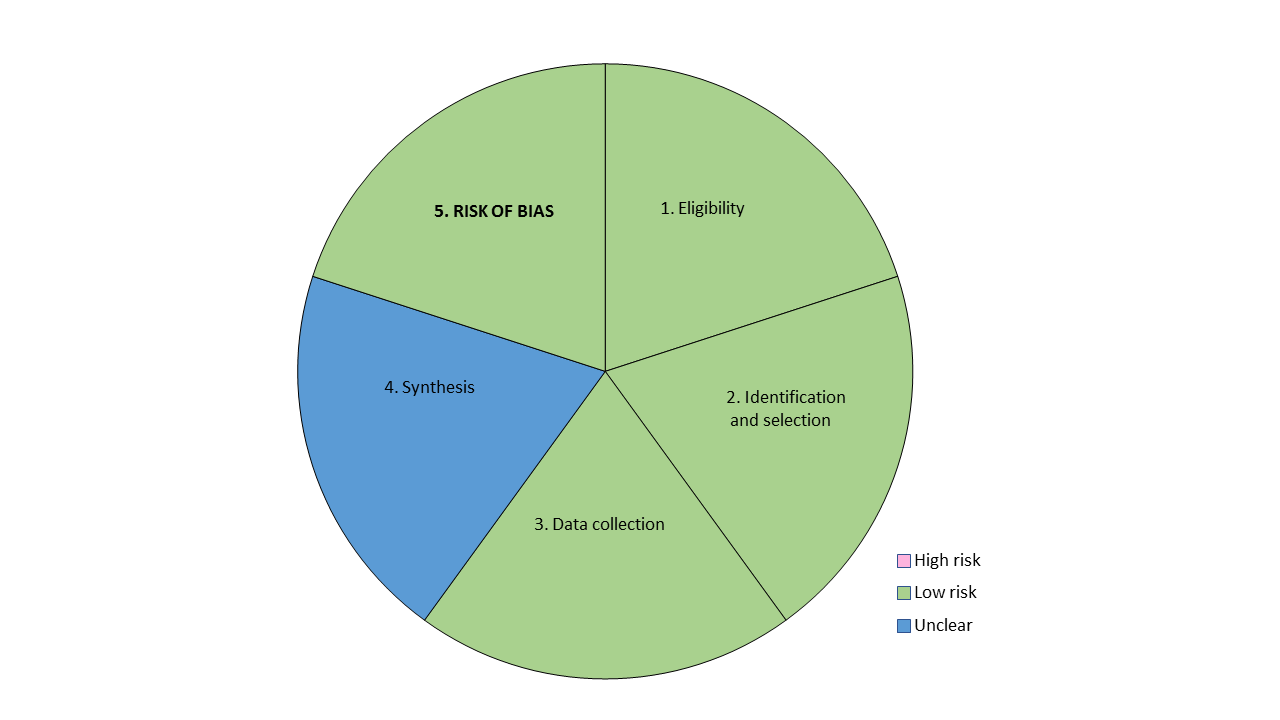

Supplement: S1 Fig — (DOCX) [file pone.0234705.s001.docx]
