# Supplementary material for: Patient-physician discrepancy in the perception of immune-mediated inflammatory diseases: rheumatoid arthritis, psoriatic arthritis and psoriasis. A qualitative systematic review of the literature
Source: PLoS One. 2020 Jun 17;15(6):e0234705. doi: 10.1371/journal.pone.0234705 (PMC7299355; doi:10.1371/journal.pone.0234705)
Supplement: S1 Table — (DOCX) [file pone.0234705.s002.docx]

Supplementary Table S1

| **TERMS AND SEARCH STRATEGY IN INTERNATIONAL DATABASES** | | |
| --- | --- | --- |
|  | **PUBMED** | **ISI-WOK and COCHRANE** |
| N. | **Terms related to the condition of interest** | |
| #1 | "Arthritis, Psoriatic"[Mesh] | "Arthritis, Psoriatic” or “Psoriasis, Arthritic*” or “Arthritic Psoriasis” or “Psoriasis Arthropathica” or “Psoriatic Arthropathy” or “Arthropathies, Psoriatic” or “Arthropathy, Psoriatic” or “Psoriatic Arthropathies” |
| #2 | "Psoriasis"[Mesh] | “Psoriases” or “Pustulosis of Palms and Soles” or “Pustulosis Palmaris et Plantaris” or “Palmoplantaris Pustulosis” or “Pustular Psoriasis of Palms and Soles” |
| #3 | "Arthritis, Rheumatoid"[Mesh] | “Arthritis, Rheumatoid" |
| #4 | “psoriatic arthritis” | |
| #5 | “psoriasis” | |
| #6 | “Rheumatoid Arthritis” | |
| #7 | #1 OR #2 OR #3 OR #4 OR #5 OR #6 | |
| N. | **Terms related to discrepancy** | |
| #8 | "Dissent and Disputes" [Mesh] | "Dissent and Disputes" or “Disputes and Dissent” or “Professional-Patient Disagreement*” or “Professional-Family Disagreement*” |
| #9 | "Attitude of Health Personnel" [Mesh] | "Attitude of Health Personnel" or “Health Personnel Attitude*” or “Staff Attitude*” or “Attitude*, Staff” |
| #10 | "disputes" | |
| #11 | "dissent" | |
| #12 | "disagreement" | |
| #13 | "discordance" | |
| #14 | "disaccord" | |
| #15 | "discord" | |
| #16 | "discrepancy" | |
| #17 | "disparity" | |
| #18 | "difference" | |
| #19 | "divergence" | |
| #20 | "dissimilarity" | |
| #21 | "variance" | |
| #22 | "misalignment" | |
| #23 | "mismatch" | |
| #24 | "dissonance" | |
| #25 | "inconsistency" | |
| #26 | #8 OR #9 OR #10 OR #11 OR #12 OR #13 OR #14 OR #15 OR #16 OR #17 OR #18 OR #19 OR #20 OR #21 OR #22 OR #23 OR #24 OR #25 | |
| N. | **Terms related to the physician and patient figures** | |
| #27 | "Physician-Patient Relations" [Mesh] | "Physician-Patient Relation*" or “Relation*, Physician-Patient” or “Physician Patient Relationship*” or “Relationship*, Physician Patient” or “Physician Patient Relation*” or “Relation*, Physician Patient” or “Doctor Patient Relation*” or “Relation*, Doctor Patient” or “Doctor-Patient Relation*” or “Relation*, Doctor-Patient” |
| #28 | “Physician-Patient” | |
| #29 | “Patient-Physician” | |
| #30 | “Doctor-Patient” | |
| #31 | “Clinician-Patient” | |
| #32 | “Patient-Clinician” | |
| #33 | “Physician-Patient Relation” | |
| #34 | “Physician-Patient Relationship” | |
| #35 | “Doctor-Patient Relation” | |
| #36 | “Doctor-Patient Relationship” | |
| #37 | (“patient” AND “physician”) | |
| #38 | (“patient” AND “doctor”) | |
| #39 | (“patient” AND “clinician”) | |
| #40 | #27 OR #28 OR #29 OR #30 OR #31 OR #32 OR #33 OR #34 OR #35 OR #36 OR #37 OR #38 OR #39 | |
| **SEARCH STRATEGY** | **#7 AND #26 AND #40** | |
| **TERMS AND SEARCH STRATEGY IN SPANISH DATABASES** | | |
| **MEDES/IBECS** | | |
| N. | **Terms related to the condition of interest** | |
| #1 | “psoriasis” | |
| #2 | “artritis psoriásica” | |
| #3 | “artritis reumatoide” | |
| N. | Terms related to discrepancy | |
| #4 | “relación” | |
| #5 | “discrepancia” | |
| #6 | “discrepancia” | |
| #7 | “desacuerdo” | |
| #8 | “disputa” | |
| #9 | “divergencia” | |
| N. | **Terms related to the physician and patient figures** | |
| #10 | “médico” | |
| #11 | “paciente” | |
| **SEARCH STRATEGY** | Due to the nature of the Spanish databases, the terms searched have been freely combined with the Boolean operator "AND" | |
