# Supplementary material for: Patient-physician discrepancy in the perception of immune-mediated inflammatory diseases: rheumatoid arthritis, psoriatic arthritis and psoriasis. A qualitative systematic review of the literature
Source: PLoS One. 2020 Jun 17;15(6):e0234705. doi: 10.1371/journal.pone.0234705 (PMC7299355; doi:10.1371/journal.pone.0234705)
Supplement: S2 Table — (DOCX) [file pone.0234705.s003.docx]

Supplementary Table S2: Discrepancy and predictor factors in immune-mediated inflammatory diseases (RA, PsA and Ps)

| Author | Disease | Discrepancy area | Measurement tools | Discrepancy definition and analysis | Results |
| --- | --- | --- | --- | --- | --- |
| Discrepancy over the assessment of disease activity in RA general patients | | | | | |
| Karpouzas et al. [20] | RA | Disease activity | PtGA and PhGA through VAS (0-10)^ | Discrepancy │PtGA - PhGA│ ≥ 3  Patient-physician concordance: Lin coefficient (0 [minimum]-1 [maximum])’. | Baseline discrepancy: 43% (PD: 31.3%; ND: 11.7%).  Lin coefficient: 0.31 (95% CI: 0.24-0.38) |
| Challa et al. [13] | RA | Disease activity | PtGA and PhGA through VAS (0-100)^ | │PtGA - PhGA│ ≥ 25 | Discrepancy: 32.5% (PD: 29.5%; ND: 2.1%).  2%, 50%, and 37% of patients in the discordant group were in remission, had mild disease activity and had moderate disease activity, respectively vs. 33%, 33%, and 22%, respectively, in the concordant group (according to CDAI criteria) (p<0.001). |
| Desthieux et al. [2] | RA | Disease activity | PtGA and PhGA through VAS (0-10)^ | Different discrepancy definition among the 13 studies included:  │PtGA - PhGA│ ≥ 3/10 (n=5);  │PtGA - PhGA│ ≥ 2,5/10 (n=2);  │PtGA - PhGA│ ≥ 2/10 (n=2);  │PtGA - PhGA│ ≥ 1/10 (n=2);  │PtGA - PhGA│ ≥ 0,5/10 (n=2) | Discrepancy: 43% (95% CI: 36%-51%; range: 25%-76%); (PD: 34%; ND: 9%). |
| Ward et al. [28] | RA | Disease activity | Patient and physician: PtGA and PhGA through VAS (0-100)^  Patient: Rating scale with five items describing different health states, using vertical VAS (0-100) | PtGA - PhGA discrepancy  Rating scale - PhGA discrepancy  Patient-physician concordance: Intraclass correlation index (ICC): 0 [minimum]-1 [maximum). | Mean discrepancy: lower with rating scale vs. PhGA (p<0.0001)   - PtGA - PhGA: 8.5 ± 22.4 - Rating scale - PhGA: 2.3 ± 24.0   ICC (95% CI):   - PtGA - PhGA: 0.31 (95% CI: 0.23 - 0.40) - Rating scale - PhGA: 0.39 (95% CI: 0.32 - 0.47)   Discrepancy according to disease activity: Higher concordance (ICC) at lower levels of disease.   - Remission/mild activity (DAS28 <3.2): PtGA - PhGA: 8.6 ± 20.2 (ICC: 0.49 [0.28 - 0.78]) vs. rating scale - PhGA: 3.8 ± 14.4 (ICC: 0.58 [0.28 - 0.77]); p=0.06. - Moderate activity (3.2≤ DAS28 ≤5.1): PtGA - PhGA: 10.3 ± 22.4 (ICC: 0.44 [0.36 - 0.57) vs. rating scale - PhGA: 6.3 ± 23.8 (ICC: 0.48 [0.40 - 0.58]); p=0.007. - Severe activity (DAS28 >5.1): PtGA - PhGA: 6.2 ± 22.8 (ICC: 0.16 [0.03 - 0.34]) vs. rating scale - PhGA: -2.9 ± 25.2 (ICC: 0.36 [0.26 - 0.48]); p<0.0001. |
| Smolen et al. [25] | RA | Disease activity | PtGA and PhGA through VAS (0-10)^ | Discrepancy │PtGA - PhGA│ ≥ 2 | Baseline discrepancy: PD: 25.5%; ND: 6.3%  Week 36 of etanercept + methotrexate treatment: PD: 24.8%; ND: 2.4 % |
| Predictor factors of (higher) discrepancy in RA general patients | | | | | |
| Karpouzas et al. [20] | RA | Disease activity | Multinomial logistic regression (PD or ND vs. concordance) |  | - Predictors of PD (baseline visit): higher fatigue, pain, HAQ-DI, lower TJC and SJC, and worse GH; (p<0.02). - Predictors ND (baseline visit): lower pain, higher TJC and SJC and PHQ-9 (p<0.01). |
| Challa et al. [13] | RA | Disease activity | Multivariable logistic regression |  | - Diagnosis of fibromyalgia (adjusted odds ratio (OR) 3.06; 95% CI: 1.87- 8.00) - Depression (adjusted OR: 1.79; 95% CI: 1.02 - 3.15) - Lack of articular erosions (adjusted OR: 0.56; 95% CI: 0.32 - 0.97). |
| Desthieux et al. [2] | RA | Disease activity | Systematic review and metaanalysis. Predictors of discrepancy collected in each study. |  | - Pain (n=5, 100% show positive association) - TJC/SJC (n=6; 67% show positive association) - Higher levels of depressive symptoms (n=1) - Health literacy (n=1) |
| Smolen et al. [25] | RA | Disease activity | Pearson’s *r c*orrelation.  Logistic regression (discordant group vs concordant group). Odds ratio (OR) |  | Baseline factors correlated with 36-week discrepancy (r<0.25, p<0.0001 to p<0.05)   - Directly correlated: BPI, duration of morning stiffness and GH. - Inversely correlated: fatigue, and SJC.   Factors measured in week 36 correlated with the discrepancy:   - Weak correlation (r<0.25, p<0.0001): directly correlated: DAS28, duration of morning stiffness, HAQ-DI, CDAI y SDAI; inversely correlated: fatigue. - Moderate correlation: BPI, GH (r= 0.48 y r=0.58, respectively, p<0.0001).   Factors measured at baseline predicting the discrepancy at week 36 (OR, 95% CI):   - BPI 1.22 (1.11 - 1.35), CRP 0.98 (0.97 - 1.00) and GH 1.02 (1.00 - 1.03). |
| Discrepancy over the assessment of disease activity in RA patients in remission | | | | | |
| Smolen et al. [25] | RA | Disease activity | PtGA and PhGA through a numerical rating scale (0-10)^ | Percentage of discrepancy according to the activity remission criteria considered:  Clinical remission: SJC y TJC ≤1 y CRP ≤1 mg/dL.  Remission according to Boolean criteria: PtGA ≤ 1  Remission according to CDAI index: CDAI ≤ 2.8 | At baseline, 58.5% of patients met clinical remission criteria (SJC and TJC ≤ 1 and CRP ≤ 1 mg/dL), and only 33.8% of total patients also met Boolean remission criteria (PtGA ≤ 1); while 26.5% of patients reached remission according to CDAI (≤ 2.8).   - Discrepancy in patients with SJC and TJC ≤1 and CRP ≤1 mg/dL and PtGA ≤ 1: PD: 0 %; DN: 2.0 %. - Discrepancy in patients with SJC and TJC ≤1 and CRP ≤1 mg/dL and PtGA > 1: PD: 49.2%; DN: 1.1%. - Discrepancy according to CDAI: PD: 7.8 %; DN: 1.0 %. |
| Wolfe et al. [30] | RA | Disease activity | Specific remission questionnaire (“Given all your experience with disease activity in RA, are you [is your patient] currently in remission?”) | Patient in remission by physician assessment and not in remission by patient assessment, or vice versa  Concordance of responses: kappa index (0 [minimum]-1 [maximum]) | Patients discordant with their physicians: 21.4%.  Patients concordant with their physicians: 78.6% (K: 0.54, 95% CI: 0.45-0.58) |
| Janta et al. [19] | RA | Disease activity | TCJ and SJC evaluated by physician and patient. DAS28 and SDAI calculation according to patient and physician data. Remission criteria: DAS28 <2.6; SDAI <3.3. | Quantitative variables comparisons (DAS28 and SDAI): Wilcoxon test. Significant differences: p≤ 0.05.  Patient-physician concordance: ICC (0 [minimum]-1 [maximum]) | Patient-physician discrepancy regarding the percentage of patients in remission:   - According DAS28: 26.1% (patients) vs. 52.2% (physicians); p<0.0005. - According SDAI: 14.5% (patients) vs. 11.6% (physicians); p=0.172.   Patient-physician concordance (ICC):   - DAS28: 0.620 - SDAI: 0.678 - TJC: 0.509 - SJC: 0.279 |
| Predictive factors of discrepancy in RA from patients’ perspective | | | | | |
| Kvrgic et al. [22], | RA | Disease activity | Semistructured interviews. | Factors reported by patients | - Being misunderstood by others - Limitations of physician assessments - Discrepancy with physicians’ findings - Inadequate active listening by doctors - Unmet psychosocial needs - Lack of patient empowerment during clinical visits |
| Walter et al. [27], | RA | Disease activity | Focus group interviews of patients with PD with their physicians. | Factors reported by patients | Predictor factors: A clear cause of discrepancy is not identified, but the following factors can contribute to the higher perception of disease activity in patients than physicians:  1) perceived stress, 2) balancing activities and rest, 3) medication intake, 4) social stress, 5) relationship with professionals, 6) comorbidity, and 7) physical fitness. |
| Discrepancy and predictor factors over RA treatment | | | | | |
| De Mits et al. [15],  2016 | RA | Treatment | Likert scale (0-10) | Score ≥ 9 on the Likert scale is considered good satisfaction. | Satisfaction with symptom control:   - 44% of satisfied patients vs. 35% of satisfied physicians [OR = 3.9 (2.6 ± 5.8); p < 0.001].   Satisfaction with route of administration:   - IV route: 52.4% of satisfied patients vs. 29.9% of satisfied physicians (p<0.001). - SC route: 56.2% of satisfied patients vs. 45.5% of satisfied physicians (p<0.001). |
| Markenson et al. [24], 2013 | RA | Treatment | PtGA and PhGA through Likert scale (0-10)^ | Cohen's kappa coefficient (κ) (0 [minimum]-1 [maximum])* | Baseline assessment: similar PtGA and PhGA scores (5.90 vs. 5.85)  5 years follow-up: PtGA higher than PhGA scores (between 4.05 - 4.46 for PtGA vs. 2.74 - 3.76 for PhGA).  PtGA and PhGA correlation remained stable over the 5 years follow-up (κ at baseline assessment: 0.49 [95% CI 0.46 - 0.51]; κ at 6 months: 0.54 [95% CI 0.51-0.57]; κ after 5 years: 0.45 [95% CI 0.41-0.50). |
| Discrepancy over clinical visit expectations in RA | | | | | |
| Wen et al. [29], 2012 | RA | Clinical visit expectations | Expectations of patients and physicians about what was most important to achieve during a rheumatology clinic visit. | Specific questionnaire | Patient: Main expectations: pain control (63.7%); improvement OF function (49.3%); discussion about the effect of medication (38.1%).  Physician: Main expectations: pain control (59.5%), inquiry about drug side-effects (47.8%); objective assessment of disease activity (41.4%). |
| Discrepancy over remission concept in RA | | | | | |
| Acebes et al. [12], 2017 | RA | Remission concept | Focus groups | Co-occurrence of attributes about the concept of remission | Rheumatologists: highlighted quantifiable objective parameters  Patients: preferred subjective measures of remission (need of finding a new definition of remission, new assessment tools that consider their feelings and all the symptoms they suffer) |
| Discrepancy over the assessment of disease activity in PsA | | | | | |
| Eder et al. [3], 2015 | PsA | Disease activity | PtGA and PhGA through numeric rating scale (0-10)^ | │PtGA - PhGA│>2 | Joint activity: 32.8% (PD: 31.2%, ND: 1.6%)  Skin activity: 22.2% (PD: 15.4%, ND: 6.8%) |
| Desthieux et al. [16], 2017 | PsA | Disease activity | PtGA and PhGA through numeric rating scale (0-10)^ | │PtGA - PhGA│≥3 | Discrepancy: 29.1% (PD: 25.0%, ND: 4.1%)  Discordant patients in remission: 30.8%  Discordant patients with a high disease activity: 26.1% |
| Furst et al. [17], 2017 | PsA | Disease activity | Satisfaction with PsA control (categorical question or Likert scale). | Physician satisfaction and patient dissatisfaction, or *vice versa*. | Discrepancy: 23.6% (satisfied patient- dissatisfied physician: 17.0%; dissatisfied patient - satisfied physician: 6.6%) |
| Predictor factors of patient-physician discrepancy over disease activity in PsA | | | | | |
| Eder et al. [3], 2015 | PsA | Disease activity | Multivariate regression analyses |  | Joint activity: fatigue (21.3%), TJC (16.3%), pain (9.2%), and SJC (1.5%)  Skin activity: pain (17.3%), DLQI (14%) and PASI (11.8%) |
| Desthieux et al. [16], 2017 | PsA | Disease activity | Multivariable linear regression |  | Higher fatigue, lower self-perceived coping and impaired social participation were related to a higher difference between PtGA and PhGA. |
| Furst et al. [17], 2017 | PsA | Disease activity | Multivariate logistic regression |  | SJC (p= 0.020)  HAQ-DI (p=0.025) |
| Discrepancy over treatment in Ps | | | | | |
| Korman et al. [21], 2016 | Ps | Treatment | Treatment satisfaction (specific questionnaire) | Physician satisfaction and patient dissatisfaction, or vice versa. | Discrepancy: 18,4%  In 70.4% of cases, patient was satisfied, and physician dissatisfied. |
| Daudén et al. [14], 2011 | Ps | Treatment | Treatment satisfaction and treatment compliance (specific questionnaire) | Chi-square test: p<0.05. | No significant discrepancies on treatment satisfaction and treatment compliance between physicians and patients were observed (p>0,05). |
| Gonzalez et al. [18], 2016 | Ps | Treatment | Discrete-choice experiment: to quantify preferences for improvements in Ps symptoms.  Maximum Acceptable Risk: willingness to accept risks (lymphoma risk) in exchange for a benefit (total plaque clearance) | Preferences for treatment attributes. | Patient-physician discrepancy regarding:   - Improvements in plaques on limbs were more important than plaques on the torso for physicians, but not for patients. - Patients perceived a significant benefit in reducing mild plaque area from 10% to 0%, but not physicians. - Patients perceived the impact of an area of 10% very severe plaques to be much more important than dermatologists. - Dermatologists valued improvements in very severe plaques for areas greater than 10%, but patients were insensitive to changes in the affected area beyond 10%. - Dermatologists were more sensitive to 10% lymphoma risk in the next 10 years than patients.   Maximum Acceptable Risk was higher in patients than in physicians (patients were willing to accept up to a 17% risk of lymphoma in the next 10 years to clear very severe psoriasis from the limbs vs. dermatologists a 9%). |
| Discrepancy over patient-physician relationship in Ps | | | | | |
| Daudén et al. [14], 2011 | Ps | Patient-physician relationship | Specific questionnaire on patient-physician relationship | Chi-square test: p<0.05. | No significant differences were observed:   - Almost all patients and physicians considered their relationship was good or very good (96.4% vs. 96%, respectively). - Patients had a good opinion about the physician (98% vs 95.2%) - Patients were satisfied with the treatment received (92% vs 94.7%)   Patients were satisfied with the time spent by the specialist (97.1% vs 92.2%). |
| Uhlenhake et al. [26], 2010 | Ps | Patient-physician relationship | Perception of patient-physician communication in relation to Ps and its management (focus groups). | Qualitative comparison of the perceptions of patients and physicians through a thematic analysis. | Patients: they required more information about Ps, fast-acting treatments, clear expectations, and recognition of the emotional burden.  Physicians: they consider that patients do not internalize information adequately and need more information about treatments. |
| Discrepancy and predictor factors in disease activity assessment on RA, Ps and axSpA | | | | | |
| Lindström Egholm et al. [23], 2015 | RA, Ps and axSpA | Disease activity assessment | PtGA and PhGA through VAS (0-100)^ | │PtGA - PhGA│> 20 | RA: 49% (PD: 47.1%, ND: 1.9%)  PsA: 56.5% (PD: 56.2%, ND: 0.3%)  axSpA: 48.3% (PD: 46.9%, ND: 1.4%) |
| Predictor factors of patient-physician discrepancy | | | | | |
| Lindström Egholm et al. [23], 2015 | RA, PsA and axSpA | Disease activity assessment | Patients and physicians’ demographic characteristics.  Mixed model logistic regression analyses. | NA | Patients with higher discrepancy: Female gender, higher fatigue, higher pain, higher functional disability; Physicians: None.  RA (higher discrepancy): patient female sex, older age, lower SJC and higher TJC, CRP, treatment with biologics  PsA (higher discrepancy): lower SJC and higher TJC  AxSpA (higher discrepancy): patients’ female sex, treatment with biologics |

NA: do not apply; PtGA: Patient Global Assessment; PhGA: Physician Global Assessment; PD: Positive Discrepancy; ND: Negative Discrepancy; TJC: Tender Joint Count; SJC: Swollen Joint Count; DLQI: Dermatology Life Quality Index; PASI: Psoriasis Area Severity Index; CRP: C-Reactive Protein; HAQ-DI: Health Assessment Questionnaire Disability Index; * κ = 0, no agreement; minimum to κ = 1, total agreement, maximum; ^Higher PtGA and PhGA denote worse assessments; ‘ Lin coefficient = 0, no agreement, minimum; to 1, total agreement, maximum.
