# Supplementary material for: Patient-physician discrepancy in the perception of immune-mediated inflammatory diseases: rheumatoid arthritis, psoriatic arthritis and psoriasis. A qualitative systematic review of the literature
Source: PLoS One. 2020 Jun 17;15(6):e0234705. doi: 10.1371/journal.pone.0234705 (PMC7299355; doi:10.1371/journal.pone.0234705)
Supplement: S3 Table — (DOCX) [file pone.0234705.s004.docx]

Supplementary Table S3

| **AUTHOR** | **TITLE** | **EXCLUSION CRITERIA** |
| --- | --- | --- |
| Akhavan et al. | Discrepancy between patient and physician global assessments over time in early rheumatoid arthritis | Congress abstract |
| Akhavan et al. | Factors affecting the discrepancy between physician and patient global assessment of disease activity in early and established rheumatoid arthritis patients- results from the Ontario best practices initiative | Congress abstract |
| Barton et al. | Patient-Physician Discordance in Assessments of Global Disease Severity in Rheumatoid Arthritis | Articles included in the systematic review selected |
| Bergstra et al. | Influence of the difference between patient and physician global assessment on disease activity status in high and lower income countries: data from the meteor database | Congress abstract |
| Cho et al, | What factors affect discordance between physicians and patients in the global assessment of disease activity in rheumatoid arthritis? | Non-European or North American population |
| Davis et al. | Pain characteristics among patients with rheumatoid arthritis in the context of patient-physician discordance in disease activity assessments | Congress abstract |
| Davis et al. | Patient-physician discordance of disease activity assessments predicts inadequate treatment response in early rheumatoid arthritis | Congress abstract |
| Davis et al. | Prevalence and correlates of patient-physician discordance in early rheumatoid arthritis | Congress abstract |
| Díaz-Correa et al. | Discordance of patient-physician assessments of general health in a us hispanic population with rheumatoid arthritis | Congress abstract |
| Feinberg et al. | Patient reported outcomes reveal discordance between Patient and Physician assessment of disease burden in patients with Rheumatoid Arthritis | Congress abstract |
| Furu et al. | Discordance and accordance between patient’s and physician’s assessments in rheumatoid arthritis | Articles included in the systematic review selected |
| Griffiths et al. | Discordance between patients with psoriasis and their physicians in assessing disease severity, psoriasis-related symptoms and disease control: Results from a large observational physician and patient survey | Congress abstract |
| Hirsh et al. | Health Literacy Predicts the Discrepancy Between Patient and Provider Global Assessments of Rheumatoid Arthritis Activity at a Public Urban Rheumatology Clinic | Articles included in the systematic review selected |
| Kaneko et al. | Determinants of Patient’s Global Assessment of Disease Activity and Physician’s Global Assessment of Disease Activity in patients with rheumatoid arthritis: A post hoc analysis of overall and Japanese results from phase 3 clinical trials | Non-European or North American population |
| Kaneko et al. | Discrepancy between patient and physician in assessment of global severity in early rheumatoid arthritis | Congress abstract |
| Karpouzas et al. | Determinants of patient- physician discordance in assessment of global disease activity in latinos with rheumatoid arthritis in the united states | Congress abstract |
| Karpouzas et al. | Rates and determinants of persistent patient-physician discordance in global assessment of disease activity in latinos with rheumatoid arthritis in the united states | Congress abstract |
| Khan et al. | Determinants of Discordance in Patients’ and Physicians’ Rating of Rheumatoid Arthritis Disease Activity | Articles included in the systematic review selected [2] |
| Kottak et al. | An Ethnographic Observational Study of the Biologic Initiation Conversation Between Rheumatologists and Biologic-naïve Rheumatoid Arthritis Patients | The main objective does not correspond to the objective of the review. |
| Okubo et al. | Analysis of Treatment Goal Alignment between Japanese Psoriasis Patients and their paired Treating Physicians | Non-European or North American population |
| Studenic et al. | Discrepancies Between Patients and Physicians in Their Perceptions of Rheumatoid Arthritis Disease Activity | Articles included in the systematic review selected |
| Tascilar et al. | Discrepancy between patients and physicians on global disease assessment of RA and its determinants: an analysis from the TRAV cohort | Congress abstract |
| Watanabe et al. | Discordance between patients and physicians in their assessment of rheumatoid arthritis disease activity | Congress abstract |
