# Supplementary material for: Patient-physician discrepancy in the perception of immune-mediated inflammatory diseases: rheumatoid arthritis, psoriatic arthritis and psoriasis. A qualitative systematic review of the literature
Source: PLoS One. 2020 Jun 17;15(6):e0234705. doi: 10.1371/journal.pone.0234705 (PMC7299355; doi:10.1371/journal.pone.0234705)
Supplement: S4 Table — (DOCX) [file pone.0234705.s005.docx]

Supplementary Table S4

| Author, year | Study design | Selection | | | | Comparability | Outcome | | Result |
| --- | --- | --- | --- | --- | --- | --- | --- | --- | --- |
|  |  | **Representativeness of the sample** | **Sample size** | **Non-respondents** | **Ascertainment of exposure** | **Based on design and analysis** | **Assessment of outcome** | **Statistical test** |  |
| Acebes et al. [12], 2017 | Cross-sectional |  |  |  |  |  |  |  | 0^∫^ |
| Challa et al. [13], 2017 | Cross-sectional | * | * | * | ** | ** | ** | * | 10 |
| De Mits et al. [15], 2016 | Cross-sectional | * |  | * | * | * | ** | * | 7 |
| Janta et al. [19], 2013 | Prospective and cross-sectional |  |  | * | ** | ** | ** | * | 8 |
| Karpouzas et al. [20], 2017 | Prospective  (2-year follow up) | * |  | * | * | ** | ** | * | 8 |
| Kvrgic et al. [22], 2017 | Cross-sectional |  |  |  |  |  |  |  | 0^∫^ |
| Markenson et al. [24], 2013 | Retrospective | * |  | * | ** | ** | ** | * | 9 |
| Smolen et al. [25], 2016 | Retrospective (36-weeks follow-up) | * |  | * | ** | ** | ** | * | 9 |
| Walter et al. [27], 2017 | Cross-sectional |  |  |  |  |  |  |  | 0^∫^ |
| Ward et al. [28], 2017 | Prospective (4-month follow-up) | * |  | * | ** | ** | ** | * | 9 |
| Wen et al. [29], 2012 | Cross-sectional | * |  | * | * |  | ** | * | 6 |
| Wolfe et al. [30], 2009 | Cross-sectional | * |  |  | * | ** | ** |  | 6 |
| Daudén et al. [14], 2011 | Cross-sectional | * |  | * | * | * | ** | * | 7 |
| Gonzalez et al. [18], 2016 | Cross-sectional | * |  | * | ** | * | ** | * | 8 |
| Korman et al. [21], 2016 | Retrospective |  |  | * | * | * | ** | * | 6 |
| Uhlenhake et al. [26], 2010. | Cross-sectional |  |  |  |  |  |  |  | 0^∫^ |
| Desthieux et al. [16], 2017 | Cross-sectional | * |  | * | ** | ** | ** | * | 9 |
| Eder et al. [3], 2015 | Cross-sectional | * |  | * | ** | ** | ** | * | 9 |
| Furst et al. [17], 2017 | Retrospective | * |  | * | * | * | ** | * | 7 |
| Lindström Egholm et al. [23], 2015 | Retrospective | * |  |  | * | ** | ** | * | 7 |

^∫^qualitative studies.

NOTE: Maximum of five points for selection bias, two points for comparability bias, and three points for outcome bias; maximum score: 10; minimum score: 0; risk of bias is considered low when a study receive 7 points or more, and high when it receives 6 points or less.
